# The effects of a resistance vs. an aerobic single session on attention and executive functioning in adults

**DOI:** 10.1371/journal.pone.0176092

**Published:** 2017-04-25

**Authors:** Ayelet Dunsky, Mona Abu-Rukun, Sharon Tsuk, Tzvi Dwolatzky, Rafi Carasso, Yael Netz

**Affiliations:** 1 The Zinman College of Physical Education and Sport Sciences, Wingate Institute, Netanya, Israel; 2 Faculty of Health Sciences, Ben-Gurion University of the Negev, Beer Sheva, Israel; 3 The Hillel Yaffe Medical Center, Hadera, Israel; Texas A&M University, UNITED STATES

## Abstract

Evidence from recent studies showed that acute aerobic exercise results in improvements in different cognitive functions. The goal of this study was to assess the influence of acute bouts of aerobic versus resistance exercise on attention and executive function in adults. Thirty-nine physically active adults (age = 52±8 yr) served as participants. Each participant visited the laboratory four times: on the first visit participants performed a cognitive test (NeuroTrax) followed by an aerobic fitness assessment, as well as maximal strength test composed of six exercises. During visits 2–4, participants completed the cognitive test before and after the experimental condition, which consisted of either 25 min of aerobic exercise or resistance exercise, or watching a recorded interview show in a seated position (control condition). Findings indicated significantly higher changes in scores of attention after acute aerobic exercise (mean change 3.46, 95% CI -0.32, 7.27) than following the control condition (mean change -0.64, 95% CI -2.23, 0.96). The changes following resistance exercise (mean change -0.67, 95% CI -4.47, 3.13) were not significantly different from the changes following the control condition. Executive function scores showed a marginally significant improvement following acute aerobic (mean change 4.06, 95% CI 1.68, 6.44) and resistance exercise (mean change 3.69, 95% CI 0.78, 6.60), but not after control (mean change 0.91, 95% CI -1.21, 3.02). We suggest that adults should consider augmenting both modalities into their training routines, which may improve their cognition in addition to providing other physical benefits.

## Introduction

Life expectancy is steadily increasing, with a large aging population all over the world [1]. It has been well documented that increased age is associated with lower performance on various measures of cognitive functions in comparison to that in young adults [2]. Beginning in the twenties and continuing throughout the rest of life, there is a gradual deterioration in the functioning of the brain [3]. A reduction in specific cognitive abilities, such as processing speed, executive functions, and episodic memory, is seen in healthy individuals during the aging process, due to neuroanatomical changes [4,5].

Several publications provide compelling evidence for the benefits of chronic physical activity in general on older adults' cognitive functioning [3]. More specifically, chronic aerobic exercise was shown to improve performance across a variety of tasks involving attention and memory [6–8], while chronic resistance exercise improved memory, verbal concept formation, selective attention, and conflict resolution among seniors [9,10]. The findings of a positive effect of chronic exercise led researchers to raise questions about the effect of acute exercise–a single bout of exercise–on cognitive performance, and this has become a topic of interest for many studies in young populations [11–14] and in adult populations [15–20]. Results of these studies show that 20 minutes of aerobic exercise improved reaction time [17,19] and had some beneficial effect on cognitive flexibility [18,20], while acute resistance exercise, performed for approximately 30 minutes, showed a trend of having a positive effect on executive functions [15,16].

Based on reviews and meta-analyses [21–23], it appears that the positive influences derived from acute exercise on cognition appear mainly in tasks requiring greater attention as well as executive function demands. These functions involve higher-order cognitive abilities that include multiple aspects of cognition, such as planning, updating, scheduling, and initiation [14]. These abilities are considered to be processed mainly in the prefrontal cortex, which undergoes a deterioration with aging. Thus, it is reasonable to suggest that exercises which positively affect the prefrontal cortex may improve attention and executive function among adults [24–26].

Aerobic exercise was shown to increase heart rate levels and, as a consequence, to increase arousal levels [27] and activation of a specific cortical area [28,29]. These changes were suggested to be a mechanism that mediates the relationship between acute aerobic exercise and cognition [27]. As resistance exercise was shown to increase heart rate levels [27] as well as the concentration of several hormones [30] that were correlated with cognitive improvements [31], it is reasonable to compare the effect of acute aerobic exercise versus resistance exercise on cognitive functions [14,32]. Pontifex and Hillman [14] compared the effect of acute aerobic exercise versus resistance exercise on cognitive performance among young adults. They found improvement in the speed processing of a working memory task, which represents an executive function, following acute aerobic exercise but not resistance exercise. However, they did not evaluate other executive functions, such as selective attention and inhibitory control. In contrast, Alves et al. [32] found that both acute aerobic exercise and resistance exercise improved speed processing and inhibition control in the Stroop test, which represents both attention as well as executive function, in middle-age women. However, they did not find improvement following either type of exercise in inhibition function in the Trail Making Test, which represents a different executive function. The authors suggested that the benefits of acute exercise might be task dependent.

The inconsistent as well as limited results about the different effects of acute aerobic exercise versus resistance exercise on attention and executive function of adults has raised the need for further exploration. Thus, the purpose of the current study was to extend the knowledge on the effect of an acute bout of moderately intense exercise on cognitive functions in middle-aged adults, by comparing two exercise modalities: aerobic and resistance. The primary outcomes of the study were attention and executive function scores as were evaluated by performances on three neuropsychological tests. In addition, based on the possible effect of exercise intensity on cognitive function, changes in heart rate during each one of the intervention conditions were also evaluated.

Based on the literature reviewed, our hypothesis was that resistance exercise performed at similar intensities as aerobic exercise, would lead to improvements in both attention as well as executive function in middle-aged adults.

## Materials and methods

### Participants

Thirty-nine (10 females and 29 males) physically active adults (age = 52±8 yr) served as participants. They were recruited from the general community by ads that were distributed on Facebook and in several sports clubs, and were answered by potential participants. Inclusion criteria were: non-smoking, no neurological or psychiatric disease, no prescribed medications that might alter cognitive function, and no head injury or long-term hospitalization in the previous three months. In addition, participants had to be able to perform and complete a maximal exercise test, and had to be involved in habitual physical activity at least twice a week, for at least three months before the commencement of the study. Exclusion criteria included a score of *<*24 on the Mini Mental State Examination (MMSE) [33], depression–>8 on the short version of the Geriatric Depression Scale (GDS) [34], inability to use a computer (due to difficulties in vision or motor function), and abnormal cardiac signs or symptoms (detected in the maximal exercise test).

All participants provided written informed consent for participation in the study, and the study was approved by the Ethics Committee of the Hillel Yaffe Medical Center (Hadera, Israel).

### Aerobic measurements

Participants performed a graded, progressive, maximal exercise test on a motorized treadmill (Woodway, Germany) in order to assess their aerobic fitness level (predicted VO_2_max) [35] and maximal heart rate (HR max), so that the predetermined level of intensity for the intervention session would be established individually. During the test, electrocardiogram (ECG), heart rate (HR), blood pressure, and rating of perceived exertion (RPE) were continuously monitored, using a 12-lead ECG, a sphygmomanometer, and the Borg scale [36], respectively.

### Strength assessment

Participants were assessed on six exercises: chest press, leg press, front pull down, seated rowing, seated squat, and shoulder press, using SportWorld multistation facilities (SportsWorld Ltd.). Because of the age of the participants and in light of concerns that a participant might be injured if he or she were to perform a true 1RM, the 1RM for each of the six exercises was assessed using a predicted 1RM approach. In accordance with this approach, participants were instructed to perform a single lift of a dumbbell (or more than one dumbbell) to identify the weight of a dumbbell that they felt comfortable lifting for 5–15 repetitions. Once a comfortable weight was identified, the participant lifted the dumbbell for 5–15 repetitions. The weight and the number of repetitions were then used to estimate the participant's 1RM for each exercise [37]. Based on that estimation, an individual level of intensity for the resistance intervention session was established. This approach is well published and considered to be in high correlation with true 1RM (r = .81–.96) [38].

### Cognitive assessment (attention and executive function)

Participants were administered three tests: *Go-NoGo test*–a timed continuous performance test during which responses are made to large colored stimuli that are any color but red; *Stroop test*–a timed test of response inhibition and set shifting modified from the well-established paper-based test. In the first phase (no interference [color]), participants choose the letter color of a general word. In the second phase (no interference [meaning]), the task is to choose the color named by a word presented in letters colored white. In the final phase (interference), participants choose the letter color of a word that names a different color; *Catch Game*–a motor planning test involving hand-eye coordination and rapid responses that requires participants to catch a falling object on the computer screen by moving a paddle horizontally so that it can be positioned directly in the path of the falling object.

These tests are part of the NeuroTrax (NeuroTrax Corp., Israel) computerized battery that utilizes novel adaptations of traditional neuropsychological tests. Previous research has shown that these tests have good concurrent validity and reliability, and are highly correlated with performance on traditional neuropsychological batteries [39]. The NeuroTrax battery is simple to use and requires no previous computer experience. Outcome parameters include four scores: accuracy (number of correct responses); reaction time (RT); standard deviation (SD) of RT; and given the speed-accuracy tradeoff, a performance index (computed as [accuracy/RT] x 100) that is computed to capture performance in terms of both accuracy and response time.

The scores of the outcome parameters of the three tests are averaged to produce two summary scores, each indexing a different cognitive domain as follows:

#### Attention

Mean RTs for the Go-NoGo test and the no-interference (meaning) phase of the Stroop test, and mean SD of RT for the Go-NoGo test. These variables are more related to attention as rapid responses are an indication that the participant is on-task and therefore appropriately attending appropriately to the stimuli [39,40].

#### Executive function

Performance indices for the Stroop test and the Go-NoGo test, and mean accuracy for the Catch Game. The performance index was deemed to reflect executive function–a broad construct that relates to the participant’s overall ability to successfully perform the task [39,40].

### Procedure

Each participant attended four sessions, which took place at least one week apart. Participants were asked not to engage in any structured exercise on the day of the testing session, and were asked not to consume caffeine for two hours prior to the session. The sessions were held in the following order:

#### Day 1 –Baseline testing

On the first laboratory visit, participants signed an informed consent form and then performed the NeuroTrax cognitive test, which lasted 15 minutes. An aerobic fitness assessment was then conducted. Based on the measured HR max and on the Karvonen equation, the Target training Heart Rate (THR) was calculated as 60% of the Heart Rate Reserve (HRR) [41]. This training intensity was suggested for cognitive improvement [21,22] and is within the THR range recommended by the ACSM [35] for health benefits and for cardiovascular improvement.

During a 30-min rest following the aerobic fitness assessment, participants completed three questionnaires (demographic, MMSE [33], and the GDS [34]), and then performed the strength assessments (see Table 1 for demographic and clinical characteristics of the participants). The rest period of 30-min given to the participants is considered to be satisfactory for energetic recovery between the aerobic and the strength assessments [37].

**Table 1:** Demographic and clinical characteristics of participants.

Variable	Men Mean (±SD) N = 29	Women Mean (±SD) N = 10	All participants Mean (±SD) N = 39
Age (years)	51 (8.5)	53 (5.8)	52 (7.8)
Height (meters)	1.76 (0.55)	1.63 (0.25)	1.72 (0.73)
Weight (Kg)	78.5 (10.72)	69.5 (10.94)	76.2 (11.36)
BMI (Kg/m^2^)	25.5 (3.09)	26.1 (4.65)	25.6 (3.50)
MMSE (points out of 30)	28.5 (1.73)	28.3 (2.50)	28.4 (1.89)
Predicted VO_2_max (ml/kg/min)	42.4 (6.86)	32.4 (4.40)	39.8 (7.68)
Maximal heart rate (beats/min)	171.4 (10.87)	166.9 (9.53)	170.3 (10.61)
Years of education	15.6 (3.39)	16 (6.16)	15.7 (4.18)
GDS (number of participants)			
No depression (score 0–2)	27	6	34
Possible depression (score 3–6)	2	4	5

#### Days 2, 3, and 4 –Experimental sessions

The order of the different experimental sessions (i.e., aerobic, resistance and control) was randomized and counterbalanced across participants to minimize any order or learning effects. During these sessions, participants completed the cognitive test before and immediately after the experimental condition, which consisted of 25 min of either aerobic exercise or resistance exercise, or watching a recorded interview show in a seated position (which was used as the control condition). During each session, HR was measured while the participants were sitting at rest 5 min prior to the commencement of each session, and was recorded throughout all sessions by a Polar HR monitor (Model A1; Polar Electro).

**The aerobic exercise session** began with a warm-up period consisting of 3 min of slow walking on a motor-driven treadmill, at a speed that elicited an HR of no higher than 95 beats/min. The speed of walking was then increased until the participants reached their predetermined THR (within 2–4 minutes), and then 25 min of walking at the THR was performed (by adjusting the slope and/or speed of the treadmill belt). At the end of the session a 3-min cool-down was performed, with the participant walking slowly to the room in which the post-cognitive test would take place.

**The resistance exercise session** was designed to provide an exercise stimulus similar in duration and intensity to the aerobic exercise. It began with the same warm-up that was performed in the aerobic session. Then participants performed the resistance session by completing three sets of 10 slow (about two seconds for full back and forth movement) repetitions at 75% of their 1RM for each of the six major muscle groups that were assessed in the first visit (i.e., chest press, leg press, front pull down, seated rowing, seated squat, and shoulder press). Participants were given a 60-sec rest between each exercise, to ensure that they were able to prepare themselves for the correct posture needed for each machine. At the end of the session, a 3-min cool-down was performed, with the participant walking slowly to the room in which the post-cognitive test would take place.

**The control session** consisted of the participants watching a recorded interview show screened on a desktop computer, in a seated position, for 25 minutes.

### Statistical analysis

As no gender differences were observed, the cognitive scores for men and women were combined, and 2-way ANOVAs (3 conditions X 2 times) were conducted.

The ANOVA was performed separately for attention scores and for executive function scores. Contrast comparisons were used for post-hoc analyses, and Cohen’s d was calculated for comparing the cognitive changes in the aerobic treatment to that of the resistance treatment and to that of the control.

Data are presented as mean±SD or mean change and accompanying 95% CI when appropriate.

## Results

S1 Table presents raw data of the study.

### Attention

No differences were found on the pre-test between scores of aerobic relative to resistance exercise (F_1,38_ = 0.02, p = 0.89), between resistance exercise relative to the control condition (F_1,38_ = 0.96, p = 0.33), or between aerobic exercise relative to the control condition (F_1,38_ = 0.36, p = 0.55).

A 2-way ANOVA (treatment: aerobic, resistance, control, time: pre-post) revealed a marginally significant treatment X time interaction (F_2,76_ = 2.49, p = 0.09). A 2-way ANOVA (treatment: aerobic, control, time: pre-post) was conducted as a post-hoc test. Results indicated a significant treatment X time interaction (F_1,38_ = 4.239; p<0.046; *d* = 0.54) indicating that changes of attention scores following aerobic exercise (mean change 3.46, 95% CI -0.32, 7.27) are significantly higher than the changes of attention scores following the control condition (mean change -0.64, 95% CI -2.23, 0.96). No differences were found between the effect of resistance exercise (mean change -0.67, 95% CI -4.47, 3.13) relative to the control condition (F_1,38_ = 0, p = 0.99), or between the aerobic exercise to the resistance exercise (F_1,38_ = 2.64, p = 0.11).

**Fig 1:**
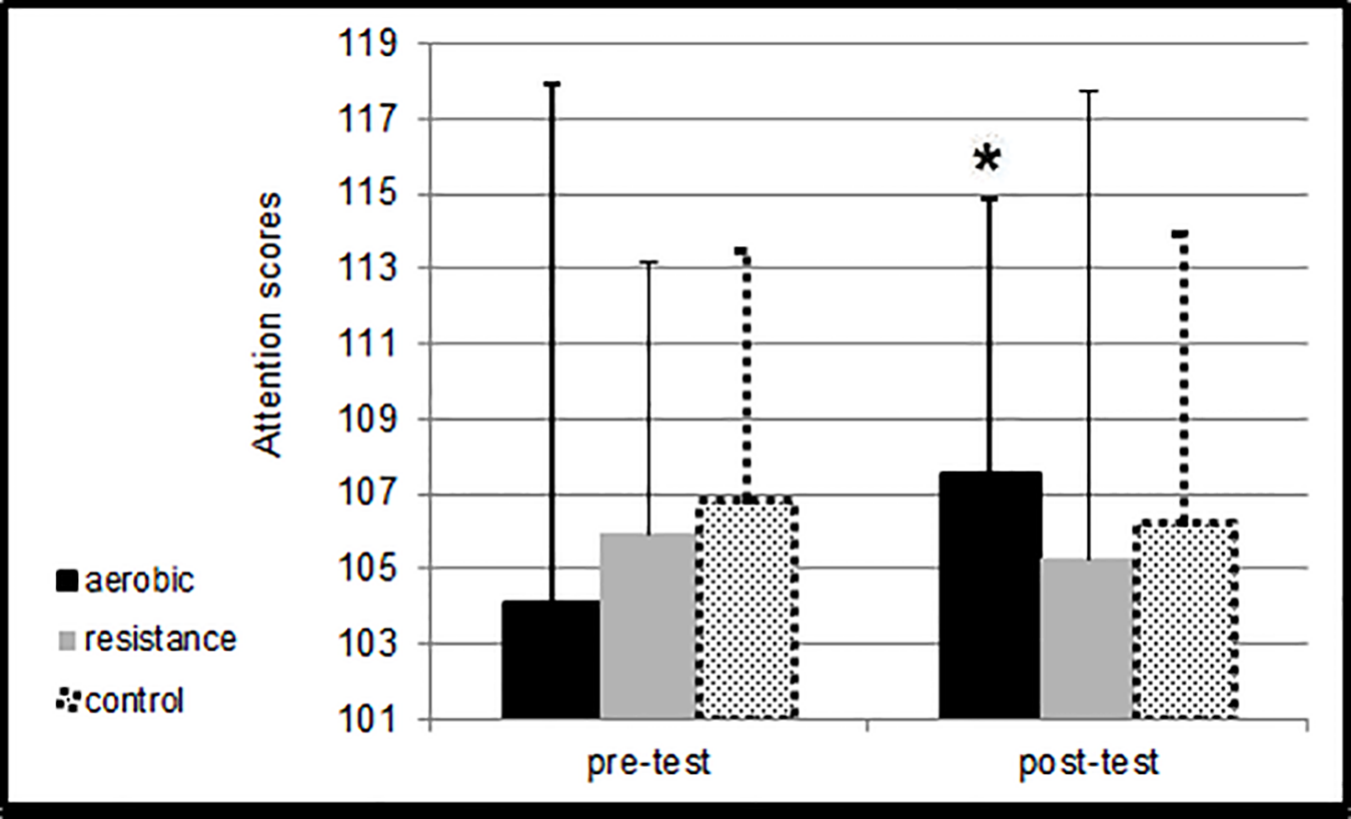
Means and SDs for the attention scores pre-test and post-test at the different sessions. * Changes of attention scores following aerobic exercise were significantly higher in comparison to score changes following control condition (p<0.05).

Fig 1 presents means and SDs for the attention scores pre- and post-intervention.

### Executive function

No differences were found on the pre-test between the scores of aerobic relative to resistance exercise (F_1,38_ = 0.22, p = 0.64), between resistance exercise relative to the control condition (F_1,38_ = 0.91, p = 0.35), or between aerobic exercise relative to the control condition (F_1,38_ = 0.23, p = 0.63).

A 2-way ANOVA (treatment: aerobic, resistance, control, time: pre-post) revealed a significant time effect (F_1,38_ = 12.497, p<0.01).

The post-hoc test revealed a marginally significant improvement in executive function scores following aerobic exercise (mean change 4.06, 95% CI 1.68, 6.44) relative to the control condition (mean change 0.91, 95% CI -1.21, 3.02) (F_1,38_ = 3.38; p = 0.07; *d* = 0.39), as well as post-resistance exercise (mean change 3.69, 95% CI 0.78, 6.60) relative to the control condition (F_1,38_ = 3.06; p = 0.09; *d* = 0.31). No differences were found between the effect of aerobic exercise relative to the resistance exercise (F_1,38_ = 0.06, p = 0.80).

**Fig 2:**
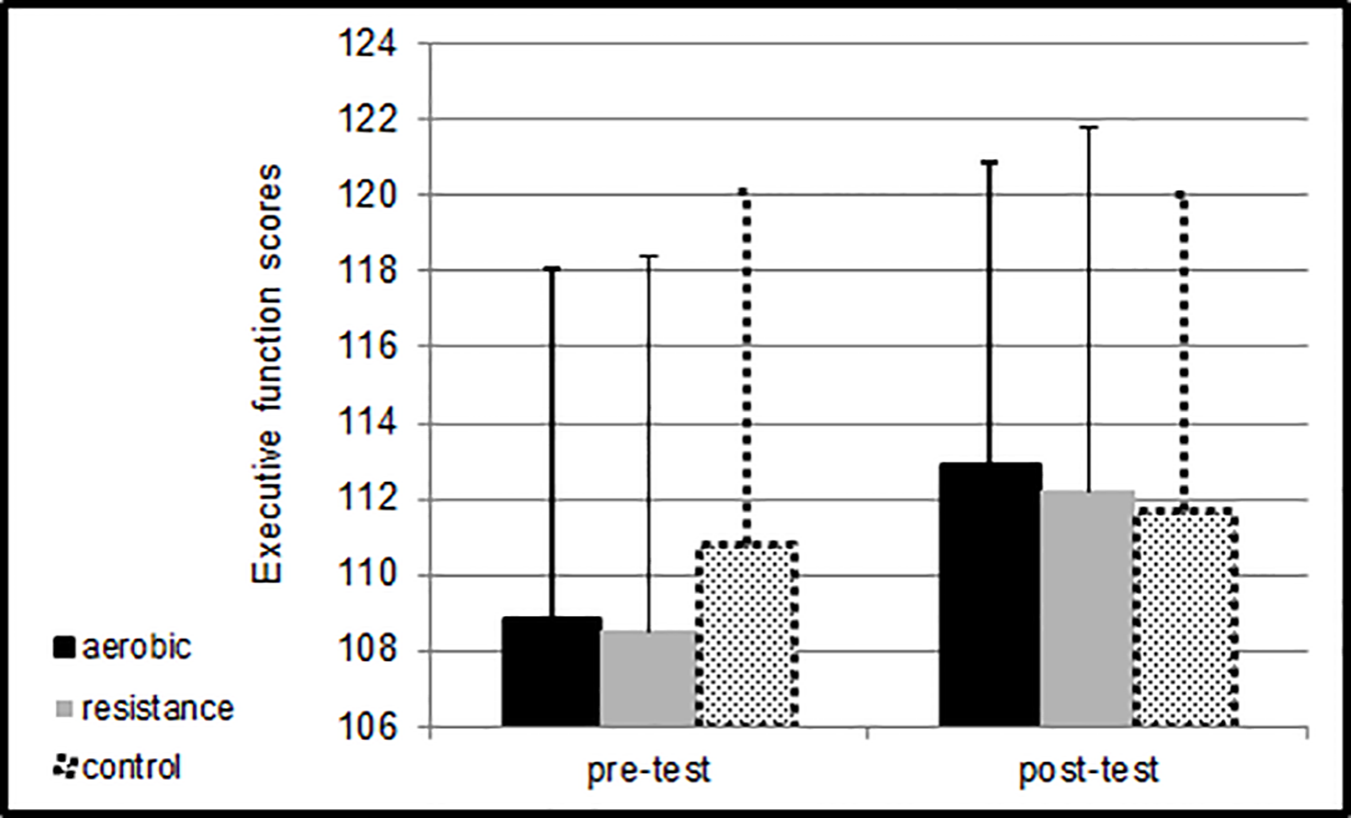
Means and SDs for the executive function scores pre-test and post-test at the different sessions.

Fig 2 presents means and SDs for the executive function scores pre- and post-intervention.

### Heart rate

Significantly higher HR was found during aerobic exercise relative to resistance exercise and to the control condition (F_2,64_ = 405.52, p<0.01). Significantly higher HR was also found post-aerobic exercise relative to resistance exercise and to the control condition (F_2,64_ = 136.21, p<0.01).

**Fig 3:**
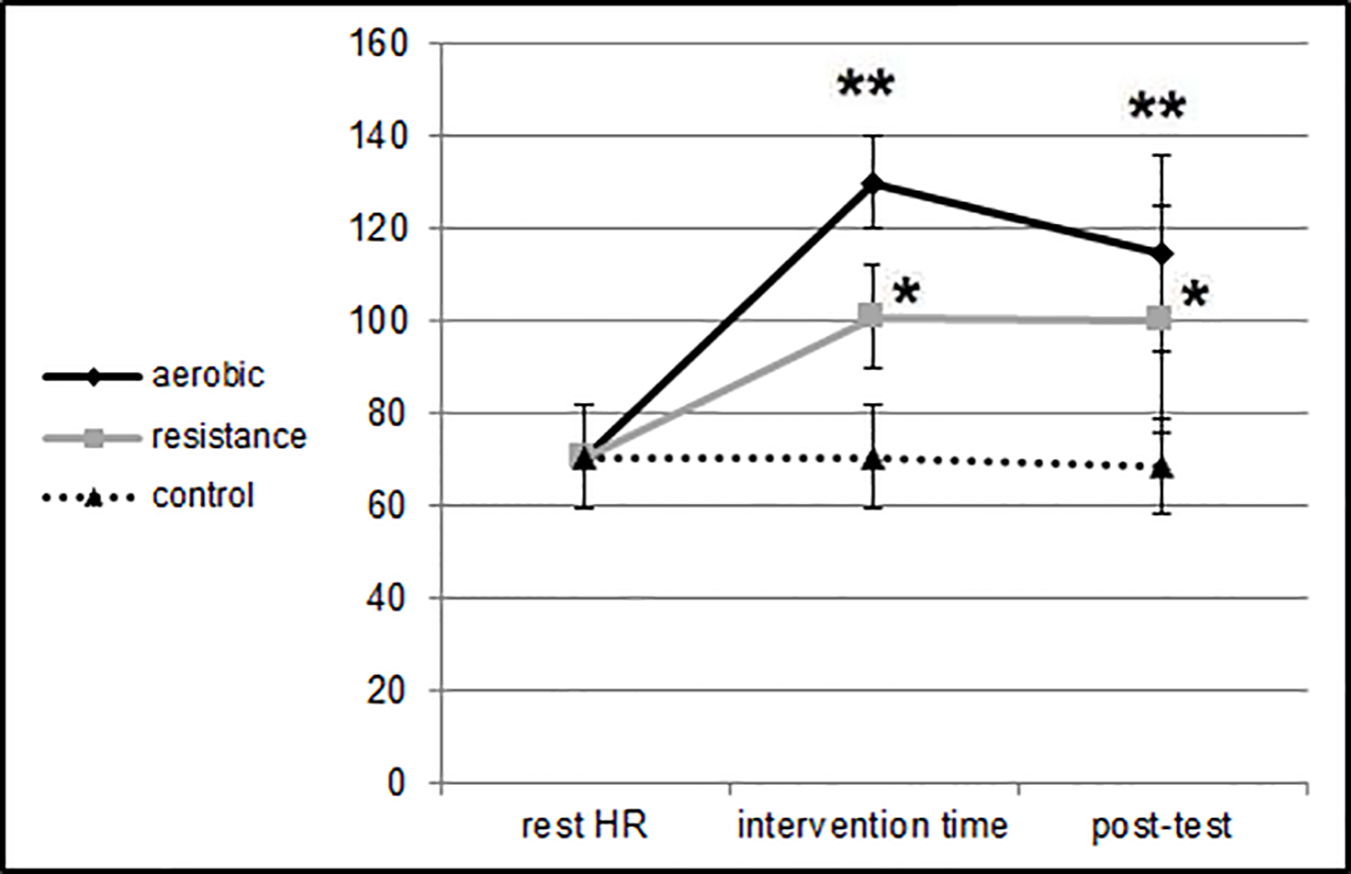
Means and SDs for the HR pre-test, during intervention, and post-test at the different sessions. ** HR during the aerobic intervention and at post-test was significantly higher than during and at post-test of the resistance intervention and the control condition (p<0.01). * HR during the resistance intervention and at post-test was significantly higher than during at post-test of the control condition (p<0.01).

Fig 3 presents means and SDs for the HR values pre-, during, and post-intervention.

## Discussion

The main result of the current study shows a positive effect of acute aerobic, but not resistance exercise, on various tasks involving attention. It should be noted, that this result was reviled by the use of post-hoc analysis, although the overall effect of both exercise modalities showed a marginally significant, however this result supports previous studies that showed improved attention following acute aerobic intervention [17–20,32,42]. The attention score in the current study was based on RT and mean SD of RT for the Go-NoGo test and on RT at no-interference phase of the Stroop test. This score is considered to represent attention, since participants must inhibit a prepotent response to read a word and activate a weaker response to name the color of the ink in which the word appears. As in our study, the RT of the Stroop test was improved following 20 min of aerobic exercise (cycling and walking) [17,19,43], while the RT of a working memory task was reduced after 15 min of moderate cycling [42], and after 30 min of moderate-intensity walking [14]. In the last study, improvements in RT were higher when the tasks placed greater demands on working memory. The authors suggested that possibly the improvement is based on the positive association found between aerobic exercise and a biochemical known to be related to neurogenesis in the hippocampus, which is involved in the completion of working memory, and possibly also in attentional demands.

Our results are partly in contrast to Alves et al. [32], who found improvement in the time to complete two conditions of the Stroop test following both aerobic and resistance acute exercise. The fact that the improvement found in the current study, as well as in the study of Pontifex et al. [14], occurred following acute aerobic exercise but not resistance exercise, may be explained by their different effect on HR. Physical activity was proposed to arouse energetic processes [29] which enhance cerebral blood flow and increase oxygen supply to neurons [44]. This enhancement has been hypothesized to alter brain systems that influence how mental resources are dedicated to cognitive task performance. This alternation is related to intensity, and can be assessed by changes in HR, oxygen uptake, RPE, or other biological indices [45]. In addition, cognitive performance was predicted to improve and peak as physiological arousal increased to optimal levels [45]. In the current study, significantly increased HR following the aerobic intervention was measured in comparison to the HR following the resistance intervention. Hogan et al. [42] found that moderate aerobic exercise at 40% to 60% of HRR led to a decrease in RT of an *n-*back task among older adults. Hogervorst et al. [43] found that a cycling exercise at an HR of 160 to 180 beats/min (which represented high activation of endurence-trained athelets) led to a decrease in RT of a stimulus-response task and of the Stoop test, suggesting improved attention. Based on these results, it is possible that the level of exercise required for improving performance of attention is higher than the level used for the resistance session in our study [46]. Accordingly, the exercise protocols for the required benefit could have been different, and this aspect should be looked into in the future.

The second result of the current study points to a general significant time effect on the executive function scores. The post-hoc analysis reviled a marginal positive effect of both acute aerobic exercise and resistance exercise on executive functions. This result is in line with the results of Chang and Etiner [16], who found a trend of improvement following resistance exercise in the Stroop test as a measure of executive function. Although not significant, the effect size for pre-post-test improvements in the current study indicated that both aerobic as well as resistance exercise had a moderate effect on this cognitive domain.

Although there is agreement that executive function refers to the ability to plan and perform goal-directed activities, and that it involves planning, scheduling, working memory, and task coordination [14,47], it has also been proposed that it is closely linked to the ability to focus on a given task necessitating attention [47]. As a result, the classification of these cognitive functions is not uniform in the literature. In our study, we used a uniform computerized battery to assess each cognitive domain separately. This battery, suggesting a classification of cognitive domains, has been used in quite a few studies in the past [48,49,50]. Consequently, attention in the present study was evaluated by means of a combination of choice RT measurements, while executive function was evaluated by a calculated formula that takes into account both accuracy and RT measures [39]. Previous studies using the Attention/ Executive function classification of the NeuroTrax found that long-term structural plastic changes in the brain were correlated with changes in executive function, among older adults, following an intervention of computerized cognitive training [48], that higher cardiovascular fitness of cardio-vascular disease patients was associated with higher scores in both attention and executive function [49], and that a single session of moderate-intensity aerobic exercise facilitate response inhibition but not motor planning or eye-hand coordination, in middle-aged healthy active adults [50]. The fact that the separation of attention and executive function revealed different effects of acute exercise in the current study may support previous suggestions about the brain’s region-specific neuronal adaptations, which are induced by various types of interventions [51]. Different cognitive tests engage different parts of the brain, and hence it is possible that the cognitive effects of a single bout of acute exercise are based on the parts of the brain that is/are activated [46,51].

### Strengths and limitations of the study

To the best of our knowledge, this is the first attempt to study the effect of acute aerobic exercise versus resistance exercise on attention and executive function among middle-aged men and women, using a randomized control approach. The results support to the assumption that resistance exercise may also stimulate cognition in middle-aged. While examining only healthy active participants may be considered a limitation of the present study, it may also provide encouragement to a large segment of healthy active people in the population by showing that aerobic and resistance exercise can enhance cognitive functioning in addition to improving physical fitness. It is recommended that the effect of acute exercise on various measures of cognition be examined in other populations, for example in people who suffer from cognitive impairments. The fact that the intensity of the resistance exercise was based on an estimation of 1RM, rather than a direct measurement, may have influenced the results. As was mentioned earlier, since direct measurements of 1RM is of high risk in the current study’s population, we could not perform them.

## Conclusions

The present findings further support the knowledge about improved attention following an acute bout of aerobic exercise. In addition, they point to a possible positive effect of an acute bout of both moderate intensity aerobic exercise and resistance exercise on executive function in middle-aged adults. Based upon this, as well as on the evidence about the importance of resistance training for older adults, these findings could be used to encourage the establishment of resistance exercise programs for adults in general.

Future research should be designed to promote further understanding of the mechanisms underlying the relationship between resistance exercise and executive function.

## Supporting information

S1 Table Raw data of demographics and results of cognitive scores at different sessions for 39 participants. (PDF)
