# Correction: The effects of a resistance vs. an aerobic single session on attention and executive functioning in adults

**DOI:** 10.1371/journal.pone.0179799

**Published:** 2017-06-12

**Authors:** Ayelet Dunsky, Mona Abu-Rukun, Sharon Tsuk, Tzvi Dwolatzky, Rafi Carasso, Yael Netz

There is an error in affiliation 2 for author Tzvi Dwolatzky. Affiliation 2 should be: The Rambam Health Care Campus and Technion, Haifa, Israel.
