# Supplementary material for: The effects of a resistance vs. an aerobic single session on attention and executive functioning in adults
Source: PLoS One. 2017 Apr 25;12(4):e0176092. doi: 10.1371/journal.pone.0176092 (PMC5404838; doi:10.1371/journal.pone.0176092)
Supplement: S1 Table — (PDF) [file pone.0176092.s001.pdf]

subject no.	gender	age (years)	MMSE	Predicted VO_2max (ml/kg/min)	Years of education	attention pre aerobic	executive function pre aerobic
1	M	42.58	30	47.30	18	111.3	116.8
2	M	64.55	30	35.70	14	105.5	111.5
3	M	45.80	30	40.20	19	111.4	110.4
4	M	43.72	30	47.30	15	96.9	101.0
5	M	54.13	29	36.50	17	106.7	111.2
6	F	48.14	29	35.70	12	51.0	81.6
7	M	61.39	30	35.70	20	112.3	116.9
8	M	48.15	29	63.40	18	109.6	116.8
9	F	59.52	30	35.70	17	108.5	107.9
10	M	41.31	30	47.30	14	113.1	115.0
11	F	45.19	30	25.50	12	100.5	94.9
12	M	64.65	30	40.25	18	112.6	109.1
13	M	70.73	29	35.70	12	111.3	114.8
14	F	58.71	29	35.00	24	101.3	107.7
15	M	65.86	28	35.00	9	109.9	109.5
16	M	58.28	29	40.00	12	104.3	109.3
17	M	56.36	28	47.30	22	98.8	106.2
18	M	44.63	30	53.50	17	102.7	109.1
19	M	50.78	29	40.30	20	108.8	109.5
20	M	41.61	30	53.50	16	107.3	113.3
21	F	58.62	29	32.00	24	119.2	122.7
22	M	52.85	28	37.80	18	113.2	124.2
23	M	53.82	24	47.30	12	101.4	95.7
24	M	42.21	27	42.40	20	113.9	115.5
25	M	57.52	27	35.70	16	109.1	110.7
26	F	54.43	30	35.70	25	99.1	101.9
27	M	44.97	29	52.10	16	97.5	104.2
28	M	42.04	27	35.70	16	48.5	81.2
29	M	53.81	29	37.80	19	109.2	118.8
30	M	43.98	30	39.90	12	100.3	111.9
31	M	45.62	26	42.40	12	105.1	100.6
32	F	54.39	26	32.20	12	105.0	107.0
33	M	51.23	29	39.90	12	107.6	119.7
34	M	45.63	26	42.40	15	111.4	111.5
35	F	43.06	29	26.00	14	112.2	112.9
36	F	54.23	24	28.30	12	107.9	114.9
37	M	49.85	27	37.80	10	103.5	99.0
38	M	42.22	25	39.90	12	107.3	111.6
39	F	54.49	29	37.80	8	104.4	108.1

attention pre resistance	executive function pre resistance	attention pre control	executive function pre control	attention post aerobic	executive function post aerobic	attention post resistance
111.990	112.9	112.3	114.4	109.1	115.5	113.980
100.090	98.0	93.1	99.0	101.8	106.3	90.910
112.680	115.3	107.4	99.0	110.2	115.8	111.340
92.430	99.9	88.4	99.8	94.8	101.6	95.950
103.760	107.8	107.1	109.7	104.8	112.8	106.710
104.380	99.5	99.8	100.4	98.9	100.3	103.850
110.940	121.1	112.3	121.9	110.8	111.3	113.630
109.450	111.7	104.5	107.6	109.7	115.3	91.720
102.600	104.8	101.8	98.7	89.4	106.1	94.380
115.540	117.5	116.4	121.0	109.3	117.3	111.780
92.990	90.0	90.4	95.7	98.6	103.8	96.270
110.740	111.7	111.5	121.3	114.7	116.3	112.540
114.030	126.5	106.1	120.3	112.3	121.7	113.810
100.170	106.8	103.5	108.1	104.7	105.3	56.690
94.840	80.1	109.4	107.8	112.5	121.6	107.350
104.790	99.2	108.1	113.9	109.9	115.0	110.020
96.090	105.3	103.3	107.1	88.0	105.9	95.620
109.260	107.6	113.1	114.0	115.1	117.7	67.490
108.770	117.1	104.7	106.7	108.6	102.8	100.890
107.750	116.3	105.2	110.3	111.6	114.7	106.380
116.050	118.8	114.3	123.9	114.0	124.2	113.450
108.020	112.9	111.1	119.6	111.5	113.9	111.260
92.140	105.7	103.9	101.4	105.4	107.5	101.790
103.630	113.1	107.8	108.2	114.0	122.2	111.490
111.460	119.5	111.0	114.8	113.2	123.6	113.300
111.270	105.8	107.9	116.8	116.8	121.9	113.380
107.690	109.9	109.5	109.8	107.8	112.0	110.990
91.370	91.3	94.0	92.0	93.0	96.7	107.200
113.460	114.1	111.2	118.5	115.4	121.2	113.070
106.380	102.4	106.8	110.8	103.9	102.9	100.980
108.480	105.1	107.7	107.8	108.8	111.0	118.980
108.670	110.7	111.5	117.9	107.4	116.1	110.390
111.230	127.6	114.7	135.2	115.6	131.9	117.460
112.400	112.3	115.2	111.6	116.0	119.4	111.350
110.300	113.4	111.0	114.6	111.0	111.7	114.090
111.490	118.2	112.8	119.9	111.2	113.9	112.240
94.070	94.8	101.4	93.1	100.4	98.7	106.870
99.670	99.6	108.0	112.5	104.1	109.0	104.550
110.780	108.8	109.4	116.2	110.2	117.8	111.400

executive function post resistance	attention post control	executive function post control	HR rest	HR during aerobic	HR during resistance	HR during control	HR post aerobic
119.1	109.6	110.1	57	121	113.4	57	97
99.8	88.3	104.2	54	117	99.4	54	86
113.4	112.7	113.4	79	129	90.1	79	108
99.8	82.8	93.7	76	129	92.8	76	93
104.1	103.5	108.7	70	125	98.5	70	95
102.2	100.2	104.7		135	104.9		131
125.2	112.9	125.1		121	99.8		124
104.4	110.0	111.6	53	125	82.4	53	77
107.0	104.1	102.0	76	118	96.2	76	95
115.1	106.4	114.2	85	144	111.3	85	114
99.3	93.5	93.1		133	105.4		142
113.2	115.9	119.9		126	107.1		130
126.5	111.9	113.3		120	103.6		117
96.1	92.3	102.3	66	124	100.4	66	92
114.6	109.4	113.2	67	122	90.9	67	122
119.6	110.5	121.9	86	135	117.3	86	125
106.8	104.1	106.0		115	80.7		97
95.9	105.2	106.8	48	126	93.8	48	76
114.4	108.7	117.3	83	138	100.7	83	113
109.2	104.3	113.9	55	124	73.9	55	66
125.0	114.5	123.0	68	128	105.9	68	120
119.8	114.1	124.0	82	144	107.9	82	132
107.9	88.5	100.6	60	124	94.6	60	125
116.0	111.4	117.3	85	147	123.2	85	139
116.1	109.1	111.4	55	122	93.3	55	87
121.5	106.2	110.1	65	115	78.0	65	83
113.0	110.6	112.8	61	130	97.9	61	126
104.6	102.0	100.6	65	120	94.6	65	115
118.5	103.9	108.9	75	128	110.0	75	126
98.0	107.4	110.6	68	128	98.9	68	120
129.5	109.4	113.2	70	142	112.2	70	141
117.0	109.7	110.4	81	128	103.5	81	125
134.8	111.7	123.2	81	147	117.3	81	124
116.0	112.9	113.3	91	154	112.3	91	146
109.8	110.1	117.3	83	147	103.6	83	141
120.9	111.0	117.7	72	123	99.0	72	120
103.1	102.7	100.7	73	140	119.1	73	134
105.4	112.1	128.3	68	135	94.6	68	130
114.1	109.1	118.0	73	138	101.8	73	135

HR post resistance	HR post control
79	66
83	53
79	75
75	61
91	70
110	
117	
53	53
87	76
90	71
144	
123	
115	
72	66
115	64
126	86
67	
64	48
87	83
55	50
123	66
127	79
109	58
146	83
73	55
57	58
116	59
87	63
127	73
100	69
115	74
98	79
127	82
114	84
100	77
100	71
122	70
121	65
111	72
